# Characterization of the Antimicrobial Edible Film Based on Grasshopper Protein/Soy Protein Isolate/Cinnamaldehyde Blend Crosslinked With Xylose

**DOI:** 10.3389/fnut.2022.796356

**Published:** 2022-02-02

**Authors:** Zisen Zhang, Xing Zhou, Changqing Fang, Dong Wang

**Affiliations:** ^1^School of Mechanical and Precision Instrument Engineering, Xi'an University of Technology, Xi'an, China; ^2^Faculty of Printing, Packing Engineering and Digital Media Technology, Xi'an University of Technology, Xi'an, China

**Keywords:** edible insects, grasshopper (*Locusta migratoria*), soy protein isolate (SPI), cinnamaldehyde (CA), antimicrobial edible film, xylose

## Abstract

A composite material based on a new insect-based grasshopper protein (GP)/soy protein isolate (SPI) blend has been studied by solution casting using xylose as a crosslinker and cinnamaldehyde (CIN) as an antimicrobial agent to develop a novel antimicrobial edible packaging. In this paper, the effects of SPI, xylose, and CIN content on the properties of edible film were studied. The tensile test confirmed that 30% SPI incorporation content had the best blending effect with the mechanical properties and barrier properties improving obviously. After adding 10% xylose to form crosslinking network, the tensile strength and elongation at the break of the film showed the best state increasing to 3.4 Mpa and 38%, respectively. The 30% CIN enabled the film to be resistant to Escherichia coli and Staphylococcus aureus strongly and decreased the water vapor permeability to 1.8 × 10^11^ (g/cm·s·Pa) but had a negative effect on the mechanical properties. This is the first time that edible insects have been used to produce the natural edible antimicrobial packaging, proving edible insects, an excellent protein source, are tipped to be a potential source of raw materials for biomaterials.

## Graphical Abstract

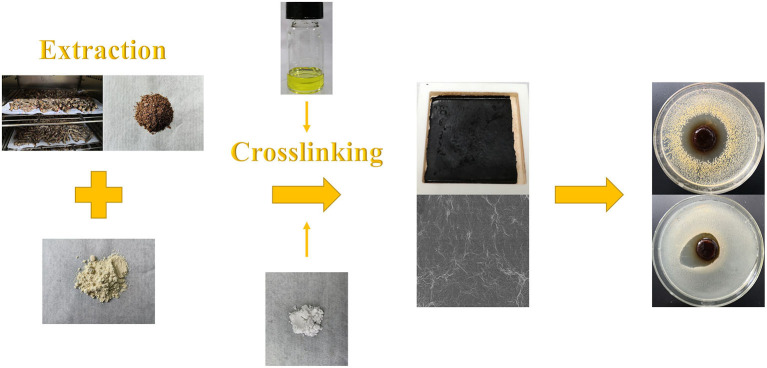

## Introduction

The design of edible films originated from addressing environmental issues caused by plastic pollution, reuse of food by-products and wastes, and consumer demands for food health and nutrition (1). Proteins (collagen, gelatin, and cereal proteins), polysaccharides (chitosan, starch, and pectin), and lipids (bee wax, and palm wax, and fatty acids) as the abundant and biodegradable resources in nature, are commonly used sources of edible films (2). Protein sources have received further attention due to their better mechanical properties than polysaccharides, good gas barriers, and can be used as a nutritional supplement (3). Currently, the most used animal protein is gelatin from beef and pork, which are not acceptable to Muslims, Jews, and Hindus for religious reasons (4). In addition, frequent worldwide outbreaks of mad cow disease have led to fears about the safety of using cattle-sourced gelatin (5). Therefore, it is necessary to find alternative protein sources.

The idea of using edible insects as an emerging protein is gaining more support from experts and public in the era of global population and cost of animal protein both growing fast and environmental problems becoming increasingly prominent (6). Edible insects are more efficient at converting feed, use less arable land, and produce fewer greenhouse gases than traditional livestock farming (7). Moreover, lower cost of technical and capital resources set a lower bar for more poor people to find jobs. Orthoptera order making up 13% of all edible insects, such as a common agricultural pest grasshopper (*Locusta migratoria*), were widely consumed in many regions around the world (8). They have a short life cycle (4–8 weeks to reach adulthood), rapid breeding rate (increase 10–16-fold in each generation), and up to 76% of the protein content (9). However, the current research on edible insects is still focused on the nutrition and processing technology of protein, and no one has explored its application as a natural macromolecule in biomaterials. If the potential value of edible insect protein used as biopolymers can be proved, and the research of edible insect protein will be extended from the application of food and feed to the biomaterials, which will develop a potential commercial market and bring substantial economic profits.

Soybean is an annual crop with a long history of cultivation and has become one of the most consumed crops in the world (10). Soy protein isolate (SPI) is the major co-product of soybean oil and it contains more than 90% storage proteins with globular structure (11). The good mechanical properties and barrier properties to oxygen and oil enabled the SPI-based film to be applicated widely in the food packaging filed (12).

Crosslinking is the formation of connecting polymer chains with covalent or non-covalent bonds to form the three-dimensional networks, such as chemical and physical crosslinking (13). The Maillard reaction releases water to form an unstable Schiff base by condensation between the carbonyl group of a sugar and the amino group of a lysine residue in a protein (14). The crosslinking network formed by Maillard reaction can effectively increase the mechanical properties and barrier properties of protein films. Saccharides such as xylose and lactose were often used as crosslinkers to improve the properties of protein films. Etxabide et al. (15) found that lactose increased the hydrophobicity and decreased the film solubility of fish gelatin. The result of the tensile strength (TS) and elongation for the xylose-modified peanut protein film markedly increased obtained by Liu et al. (16). Cinnamaldehyde (CIN) is a high effective antimicrobial agent for killing the microorganism which is the main component of essential cinnamon oil (about 85%) with high purity of 98% (17). It is also used as a green crosslinker that crosslinks with the protein under alkaline conditions *via* Schiff base and Michael's addition reaction (18).

Based on the aforementioned interest, a novel antimicrobial edible film based on the grasshopper (*Locusta migratoria*) protein (GP) has been developed for the first time in this study. The main objective of the study was to evaluate the effects of each component on the physicochemical properties of the film. Firstly, the film-forming properties of incorporating SPI into GP were studied. Then, the xylose was used as a crosslinking agent to modify the blend protein film. Furthermore, we added CIN to explore whether it could form a double crosslinking enhancement effect with xylose and maintain the antimicrobial activity of the composite film at the same time.

## Materials and Methods

### Materials

Adult grasshoppers (*Locusta migratoria*) were bought from the local farmer who breeds the edible insects. SPI was purchased from Cool Chemical Science and Technology Co., Ltd. (Beijing, China). D-xylose (98%, Mw ~150.13) was purchased from Macklin Biochemical Co., Ltd. (Shanghai, China). CIN (98%, Mw ~132.2) was purchased from Yien Chemical Technology Co., Ltd. (Shanghai, China). Glycerol was purchased from Erkang Pharmaceutical Co., Ltd. (Liuyang, Hunan, China).

### Protein Extraction

Protein extraction was followed by the typical alkaline dispersion and acid precipitation method according to a modification of the method by Mishyna et al. (8). The grasshoppers were all removed wings and dried in the oven at 40°C for 24 h. The water-removing insects were powdered with a pulverizer (XL600B, Xiaobao, China). Raw powders were dispersed in hexane keeping stirring at 1:5 (w/v) at 45°C for 1 h and then pouring the upper liquid. The same step was repeated 3 times and sedimentation was dried in an oven for 24 h to obtain the defatted grasshopper powders.

The defatted grasshopper powders were mixed with deionized water in a ratio of 1:8 and adjusted pH to 10 with an instrument (S210, Mettler-Toledo, Switzerland). The solution was stirred with mechanical agitation for 1 h at 60°C and sonicated by ultrasonic homogenizer (JY92-IIDN, Scientz, China) at 200 w for 30 min. The mixture was centrifuged at 10,000 *g* for 20 min. The pH of supernatants was adjusted to isoelectric point (4.0). Solutions were centrifuged at 10,000 g for 20 min after 5 h of static settlement at room temperature. The precipitates were redissolve in deionized water and adjusted to pH 7, then dried in freeze dryer (LGJ-25C/25E, Foring Technology, China) for 2 days to get protein powders for experiment.

### Film Preparation

Approximately 6% (w/v) GP/SPI blend (8/2, 7/3, 6/4) were dissolved in deionized water. Glycerol was added at 45% (w/w) of protein powders. Xylose was added at 5, 10, and 15% (w/w) of protein powders. The pH of solution was adjusted to 10 with 5 M NaOH solution and solution was heated under magnetic stirring (RCT basic, IKA, Germany) at 80°C for 30 min at 600 rpm. CIN was added at 10, 20, and 30% (w/w) of protein powders after solution was cooled to 40°C and stirred for another 20 min. Then pour solutions into PTFE molds (8 × 8 cm) in oven at 40°C for 48 h and peeled off. All the samples were labeled as control, 8–2, 7–3, 6–4, XY5, XY10, XY15, CIN10, CIN20, and CIN30 in the above order, respectively.

### Film Characterization

#### Scanning Electron Microscope

The section morphology of films was imaged with scanning electron microscope (SEM) (Jeol, JSM-6700, Japan) setting the accelerating voltage at 3 kV and magnification at ×5,000. The samples were fractured in liquid nitrogen and the fractured surfaces were observed after sputter-coated with gold.

#### Fourier Transform Infrared Spectroscopy

The films were scanned with an attenuated total reflectance–Fourier transform infrared (ATR–FTIR) spectrophotometer in the range of 500–4,000 cm^−1^ wavenumber (Vetex 70v, Bruker, Germany).

#### X-Ray Diffraction

The GP films were analyzed by X-ray diffractometer (Shimadzu, XRD-7000, Japan) from 5 to 40° at 4°/min with Cu Kα source at 40 kV and 40 mA.

#### Mechanical Properties

Tensile strength and elongation at break (EAB) of films were tested according to the ASTM-D882 with modifications using Electronic Strength Tester (C610M, Labthink, China). The samples (75 × 15 mm) were stretched with load cell of 500 N at a speed of 20 mm/min.

#### Thermogravimetric Analysis

Thermal stability was observed in terms of total weight losses by using the instrument (TG 209 F3 Tarsus®, NETZSCH, Germany). And the test was carried out at a heat/cooling rate of 30 ml/min in the temperature ranging from 30 to 550°C in nitrogen atmosphere.

#### Differential Scanning Calorimeter

Thermal characterization of the composite has been monitored with the instrument differential scanning calorimeter (DSC 200 F3 Maia®, NETZSCH, Germany). All the samples (~5–10 mg) were heated from 30 to 100°C with 10°C/min, kept for 1 min at 100°C to eliminate the thermal history, and then cooled down to 0 with 10°C/min under nitrogen flow. At last, they were reheated to 200 with 10°C/min.

#### Water Solubility

The method of water solubility (WS) was described by Rhim et al. (19) with modifications. The films were cut into 3 × 3 cm and dried at 80°C for 24 h. The dried films were put inside the test tubes and then placed in an oscillator to vibrate gently for 24 h at 25°C. The undissolved parts of films were filtered using gauze and dried at 80°C for 24 h to determine their weight. WS was obtained according to the equation:


$$WS=(W_{\text{i}}-W_{\text{f}})/W_{\text{i}}\;\times \;100$$

#### Water Contact Angle

The water contact angle (WCA) was tested with water contact angle measurement apparatus (OCA 20, Dataphysics, Germany) based on the sessile drop method. The droplets of 2 μl were dropped onto the sample surface at 5 different points, respectively.

#### Water Vapor Permeability

The films were cut into a circular specimen with a diameter of 8 cm. The water vapor permeability (WVP) test machine (PERME^TM^W3/060, Labthink, China) was set at 25°C and 50% RH (relative humidity) to detect the mass change every 6 h automatically according to ASTM E96/E96M-2005.

#### Antimicrobial Activity

The antimicrobial test used the inhibition zone method with Gram-positive *Staphylococcus aureus* (ATCC 6538) and Gram-negative *Escherichia coli* (ATCC 25922) as test strains according to Wu et al. (20). The bacterial suspension (~10^6^ CFU/ml) of 200 μl was evenly spread over the beef extract peptone medium. Each sample was cut into disc (12 mm diameter) and sterilized with UV light, then placed in the center of each plate. The Petri dish were incubated at 37°C and 70% RH for 24 h.

## Results and Discussion

### Scanning Electron Microscope

**Figure 1:**
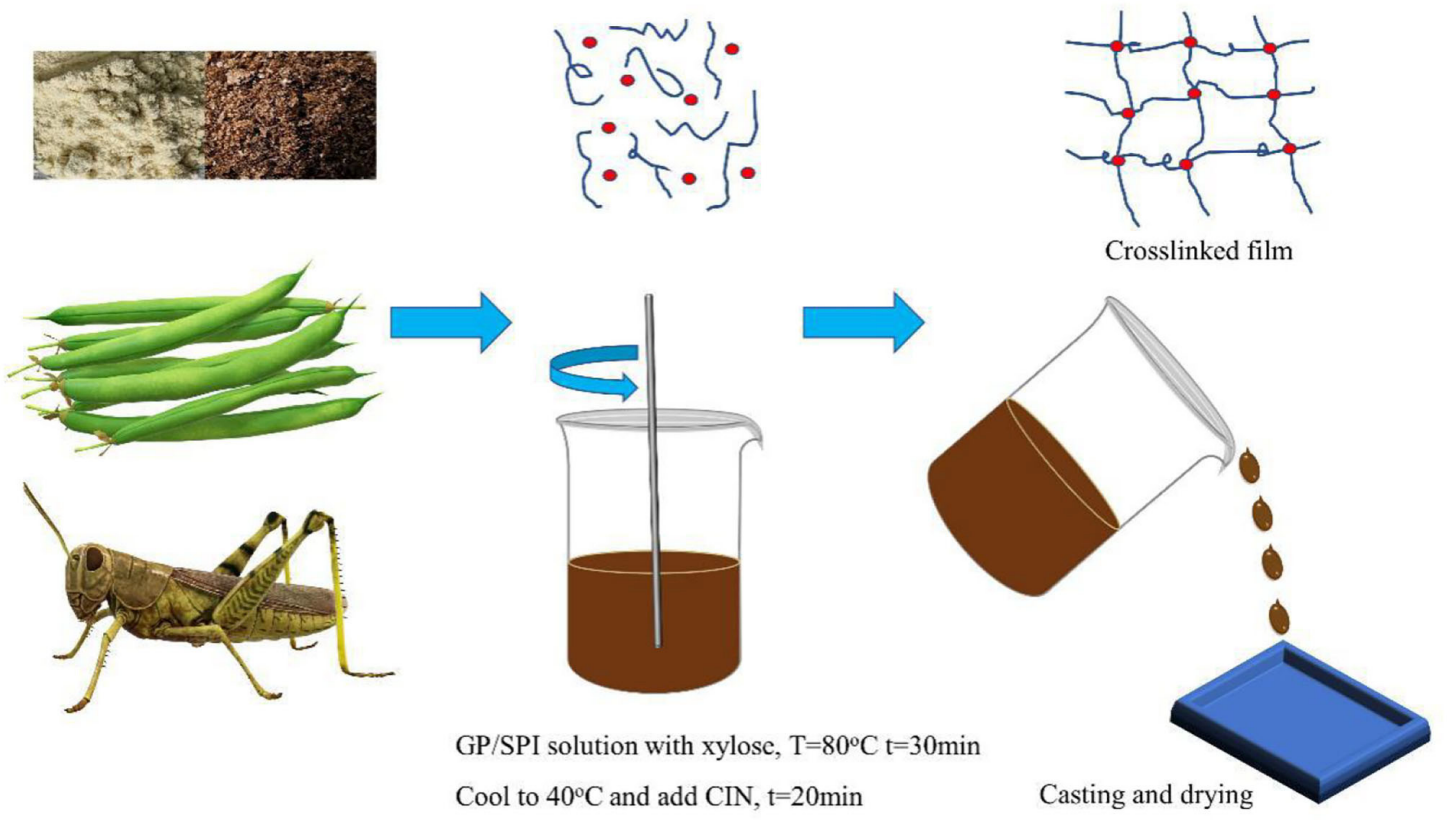
The schematic diagram of this experiment.

**Figure 2:**
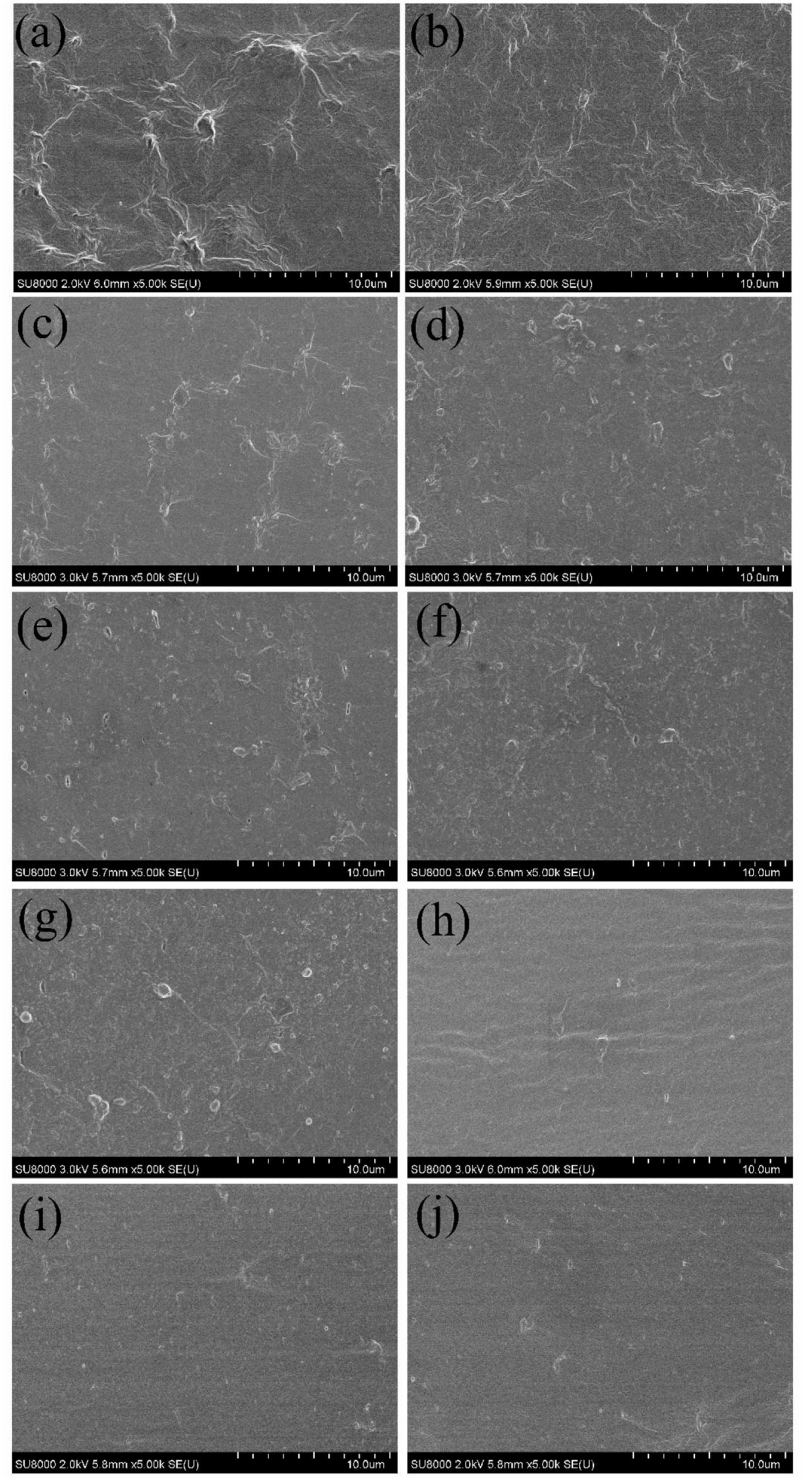
Cross-section scanning electron microscope (SEM) images for grasshopper protein (GP)-based composite films. **(a)** control, **(b)** 8–2, **(c)** 7–3, **(d)** 6–4, **(e)** XY5, **(f)** XY10, **(g)** XY15, **(h)** CIN10, **(i)** CIN20, and **(j)** CIN30.

An antimicrobial and edible composite film has been prepared on the basis of a new insect-based grasshopper protein (GP)/soy protein isolate (SPI) blend via the facile solution casting under the function of crosslinker and antimicrobial agent, as illustrated in Figure 1. As shown in Figure 1, the antimicrobial edible film has been prepared via a facile process. Cross-sectional images of all the samples are listed in Figure 2. The quenched sections of the control group surface were rugged and distributed with insoluble particles and aggregates, from which wrinkles were distributed around. Figures 2b–d shows the wrinkle-dominated morphology gradually disappeared with the addition of SPI. The quasi-circular particles of different sizes were distributed evenly on the smooth surface when the addition of SPI reached 30%. The addition of high purity commercial SPI increased the total protein content in solute and promoted the reduction of insoluble impurities. He et al. (21) observed a similar cobblestone-like surface in a SPI/wheat gluten protein complex. After xylose was added, as shown in Figures 2e,f, the particles became finer and the distribution was more uniform, forming a dense crosslinking network, which is helpful to hinder the crack propagation. The addition of crosslinker promoted the miscibility among different phases and induced a continuous and uniform matrix structure (22). The roughness, aggregates, and streaks of the fracture surface decreased with the addition of CIN, indicating fracture changes from ductile to brittle (23). There are still some micropores formed in the film drying process with the evaporation of CIN. Syafiq et al. (24) suggested that the existence of micropores could increase the volume of the film.

### Fourier Transform Infrared Spectroscopy

**Figure 3:**
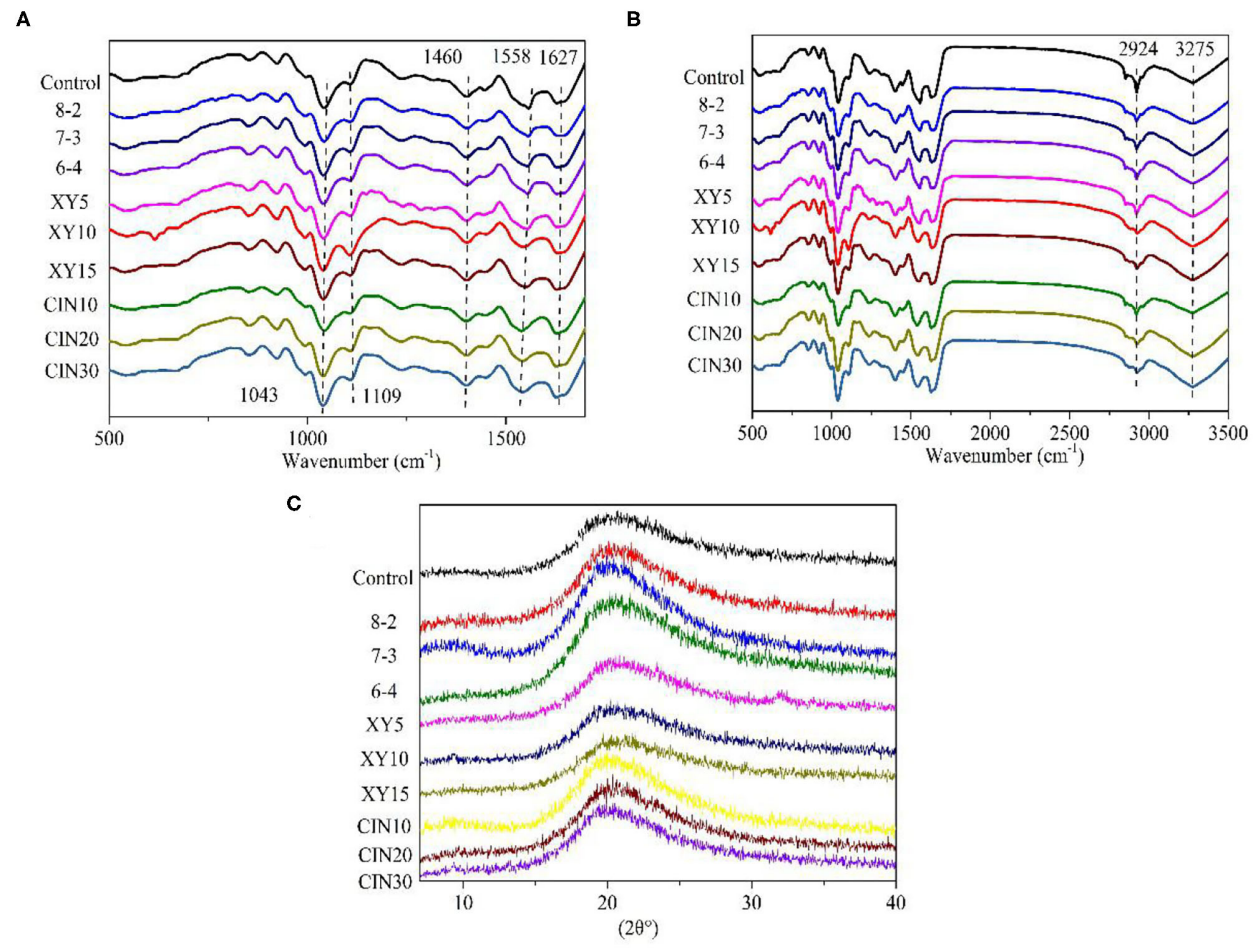
**(A,B)** Attenuated total reflectance–Fourier transform infrared spectroscopy (ATR-FTIR) spectra and **(C)** X-ray diffraction (XRD) patterns of GP-based composite films.

The ATR–FTIR spectra of films prepared from GP are shown in Figure 3. The amide I (C=O and C–N stretching vibration), amide II (N–H bending), and amide III (N–H in-plane bending with C–N stretching vibration) associated with the protein structure was distributed at ~1,627, 1,558, and 1,240–1,460 cm^−1^, respectively (25, 26). The amide I and amide II peak represented the β-sheet and α-helix structure in protein, respectively (27). The amide II peak of samples added with CIN shifted from −1,558 to −1,541 cm^−1^, suggesting inner secondary structure alternated in polypeptide chains (27). The width of amide A (O–H and N–H stretching vibration) peak at −3,275 cm^−1^ was affected by the degree of crosslinking of the protein network (25). The amide B (C–H stretching) at −2,924 cm^−1^ was related to the interaction of amino groups between peptide chains (28). The peaks at −770, −923 cm^−1^ (C–C vibrations), −1,043 cm^−1^ (C–O stretching at C1 and C3) and −1,109 cm^−1^ (C–O stretching at C2) were ascribed to glycerol (29). Compared with the control group, there was no new infrared peak in the modified protein film, which meant that no new functional groups were formed after modification. The same phenomenon was observed in several studies on protein crosslinking and protein blends (30, 31).

### X-Ray Diffraction

The X-ray diffraction (XRD) of films was analyzed by the Jade software as shown in Figure 3. The very weak peak at 9.7° represented a low level of triple helix structure. The high drying temperature is an obstacle to regenerating the helix structure in the biopolymer chain of protein (32). Moreover, the formation of crosslinking and glycoconjugates from the Maillard reaction hindered the rearrangement process of the triple helix structure and reduced the content of the triple helix structure (33). The wider hump at 20° corresponded to β-sheet structures of GP, suggesting it to be an amorphous biopolymer (34). Compared with the control group, the strength of this hump increased after the SPI addition, indicating an increase in crystallinity. The crystallinity of the film after the addition of xylose was slightly lower than that of the blend group. This was because the addition of saccharides interfered with the arrangement of the protein chain, thus reducing the crystallinity (26). The covalent bonds from the Maillard reaction also prevented the crystal nucleation and growth (35). The effect of the crosslinking reaction on the crystallinity of the protein depended on the materials and reaction conditions. Guerrero et al. (35) found the addition of lactose increased the crystallinity of SPI. Liu et al. (36) reported the degree of crystallinity of wheat protein fibers treated by microwave was higher than the untreated group. However, Li et al. (26) found that the crystallinity of peanut protein decreased after being mixed with Arabic gum. After that Wang et al. (37) treated the collagen with UV+DHT, the collagen almost lost its typical peak at 20°.

### Mechanical Properties

**Figure 4:**
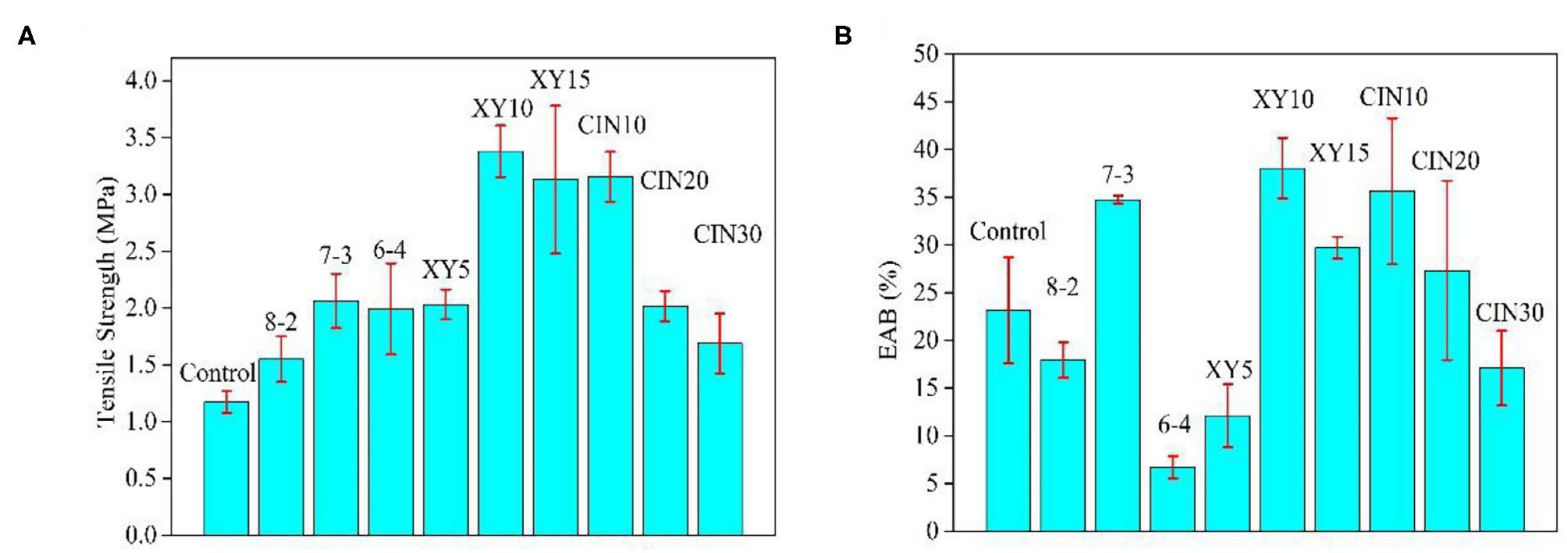
Mechanical properties of GP-based composite films **(A)** tensile strength (TS) and **(B)** elongation at break EAB.

The results of TS and EAB of composite films are shown in Figure 4. The addition of SPI significantly enhanced the TS of the composite films by the protein–protein interactions. The sequence of amino acid residues and the size of the three-dimensional network structure would determine the interactions in the end (11). The mechanical properties of the samples with 10% xylose addition were the best among all the samples, while the TS and EAB increased by 288% (from 1.2 to 3.4 MPa) and 165% (from 23 to 38%) compared with the control group. The film with xylose glycosylation was more likely to form a dense structure and high molecular weight (38). When xylose was increased from 10 to 15%, the TS and EAB decreased due to the saturation effect. Liu et al. (16) also proved this trend in his research studying the interaction of xylose with peanut protein. The CIN had a negative effect on TS and EAB, especially when the addition was more than 10%. The result of CIN on the mechanical properties of the polymer depends on the substrate, and there is no particularly consistent trend. Similar negative effects of CIN on mechanical properties were observed by Wang et al. (39) and they thought that the hydrophobic CIN molecules affect the molecular arrangement of the substrate and counteract the hydrogen bonds that had formed. Cao et al. (40) and Yang et al. (41) both reported the plasticizer effect of CIN on polymer matrix which improved the TS and decreased the EAB. Balaguer et al. (42) and López-Mata et al. (43) significantly improved the TS and EAB of the original materials by adding CIN due to its good crosslinking and plasticizer effect.

### Thermal Properties

**Figure 5:**
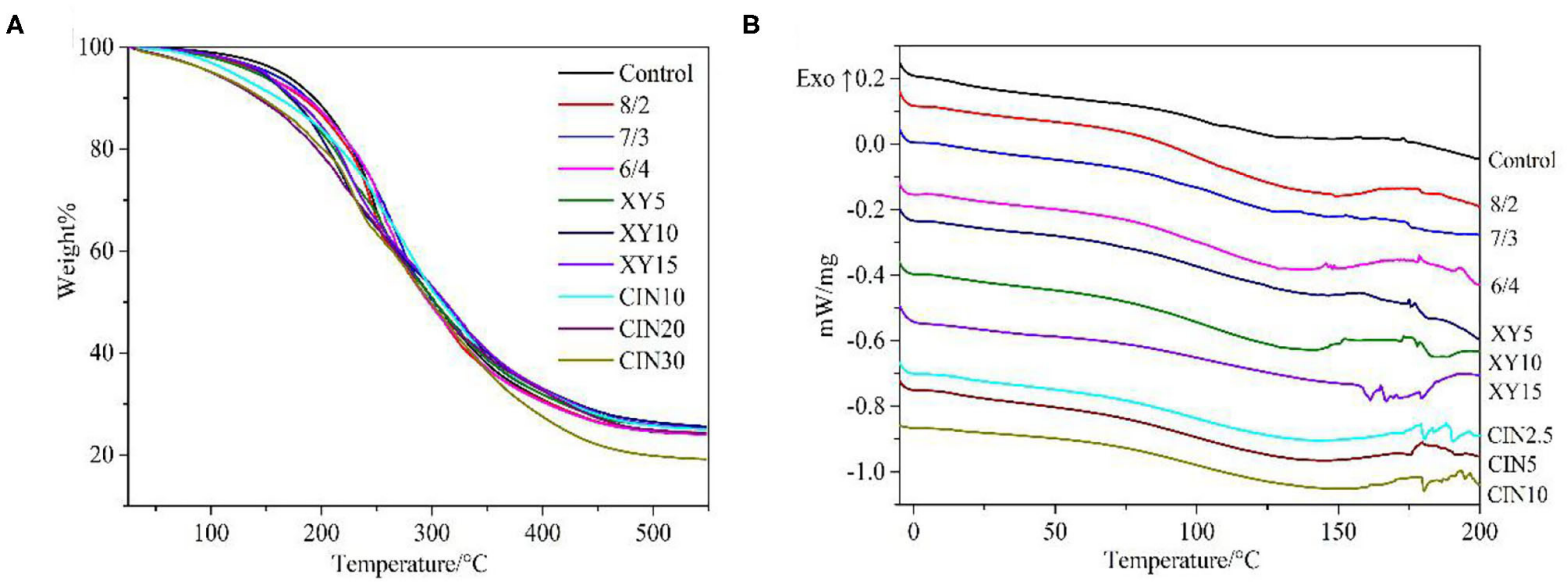
**(A)** Thermogravimetric analysis (TGA) and DSC **(B)** curves of GP-based composite films.

It can be seen from the curve in Figure 5 and from the results in Table 1 that the addition of SPI had little effect on thermal stability. The first stage of thermal degradation corresponded to the evaporation of plasticizer and water, ranging from 100 to 260°C (44). Compared with the control group, the films with addition of xylose and CIN decomposed faster at this stage, which caused by the stronger water holding capacity of the blend films after crosslinking. The thermal degradation after 260°C was mainly related to the protein matrix and involved the breaking of intermolecular hydrogen bonds within the protein (45). This result was similar to the effect of tannic acid on casein after crosslinking; both crosslinkers have a certain retarding effect on the decomposition of the protein backbone (46). Crosslinking improved the polymer thermal stability by limiting the molecular rotation and vibration that occurred during thermal excitation (47). Compared with xylose group, the 30% addition of CIN impaired the thermal stability of films. The Tg of films was slightly increased after crosslinking with xylose. Crosslinking increased Tg by reducing the mobility of the polymer chain and intramolecular free volume (48). The type of saccharide had a great influence on Tg after crosslinking. Kachou et al. (49) found that Tg decreased when they modified gelatin with glucose. Nafchi et al. (50) reported that the thermal denaturation temperature of egg white protein increased no matter adding reducing sugars or the non-reducing sugar. The addition of CIN into the crosslinked protein film led to a significant decrease in Tg. The same decreasing influence of CIN on Tg was observed in the previous studies (40, 51) and was also caused by the plasticizing effect of CIN on the matrix. Small endothermic peaks were observed between 150 and 200°C, resulting from the melting of SPI, which had a melting point at about 200°C (52).

**Table 1:** Thermal stability data for prepared films.

**Samples**	**T_**g**_ (**°**C)**	**T_**10**_ (**°**C)**	**T_**50**_ (**°**C)**	**Mass loss (%)**
Control	92.5	193.4	302.6	24.1
8/2	98	182.9	298	24.3
7/3	105.5	187.3	301.8	25.3
6/4	119	185.5	296.8	24
XY5	105.6	172	302.5	25.1
XY10	114.4	172.8	310.3	25.6
XY15	103	175	311.8	25.1
CIN10	98.1	161.1	309	25.02
CIN20	79.9	142.6	298.6	24.2
CIN30	77	146.2	298.5	19.16

### Water Solubility

**Figure 6:**
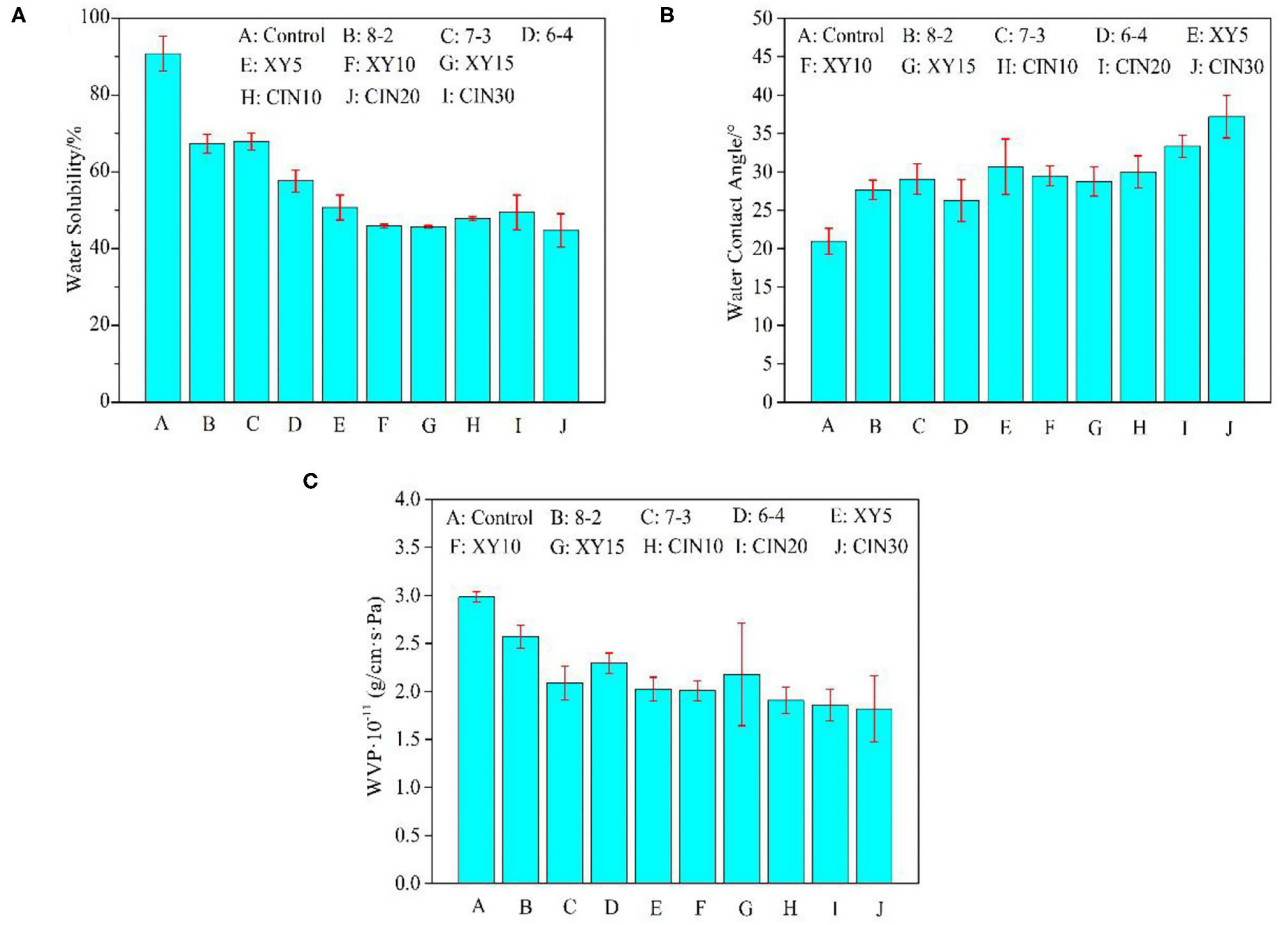
**(A)** Water solubility (WS), **(B)** water contact angle (WCA), and **(C)** water vapor permeability (WVP) of GP-based composite films.

The solubility of protein film is related to several factors, such as high interaction density (intermolecular covalent bonds or chain entanglements), isoelectric point, and ionic strength of the medium (53). The type and dosage of saccharide had a great influence on the WS after crosslinking. It can be seen from Figure 6 that WS was significantly reduced from 91 to 46% with the 10% xylose addition compared with the control group. But when the dosage reached 15%, the saturation effect appeared. The three-dimensional network formed after crosslinking had a very dense structure and a small intermolecular space, which can effectively prevent the infiltration of water molecules and reduce the WS (54). The formation of melanoidins in the Maillard reaction, the end product of glycosylation, is promoted the reduction of WS (55).

### WVP and WCA

The WCA of the films was slightly increased after blending with SPI due to the better hydrophobicity of SPI. After the addition of xylose, the WCA of the film had little change after the Maillard reaction. This suggested that the addition of xylose did not consume enough of the exposed hydrophilic groups in the protein blend (56). Liu et al. (57) also found that the increase of potassium pyroantimonate content led to the decrease of WCA when they used genipin and potassium pyroantimonate as double crosslinkers. The WCA of the composite films was further increased to 38° with 30% CIN addition because CIN can conjugate with the free amine group to replace hydrophilic groups with hydrophobic aromatic groups, thus improving the hydrophobicity of films (17).

The hydrophilicity and hydrophobicity of the material and internal voids, cracks, and torturous path structure would affect the permeability (58). As can be seen from the results in the Figure 6, WVP of protein film decreased from 3.0 × 10^−11^ to 2.0 × 10^−11^ (g/cm·s·Pa) after adding 30% SPI, which was the group of samples with the largest improvement in the blend group. The formation of crosslinking network helped to densify the structure of the film and reduced its permeability. But after the addition of xylose and CIN, the WVP showed no significant change compared with the 30% SPI group. This indicated that the effect of substrate materials was more important than crosslinker on WVP. In this paper, the influence of crosslinkers on WVP, WS, and WCA was ranked as follows: WS>WCA>WVP, which may be due to the complicated mechanism of WVP. The process of water molecules passing through polymer film can be summarized as an adsorption-infiltration-diffusion-desorption mechanism (59).

### Antimicrobial Activity

**Figure 7:**
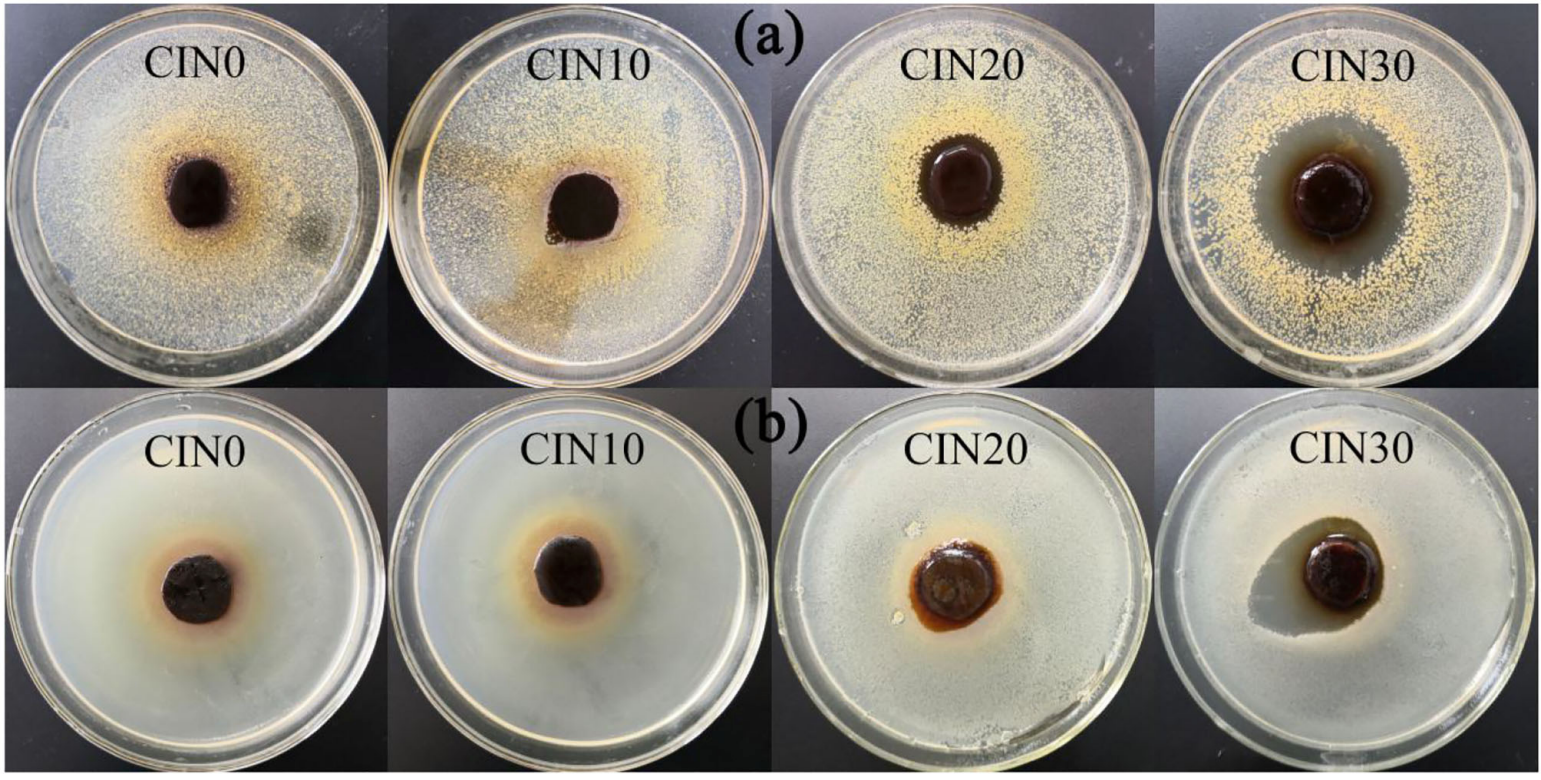
The inhibition zones of GP-based composite films against **(a)** *Staphylococcus aureus* and **(b)** *Escherichia coli*.

The results on antimicrobial ability are shown in Figure 7 and Table 2. Due to its high volatility, the composite films began to have good antimicrobial activity only when the addition of CIN reached 20%. With the increase of CIN concentration, the diameter of the inhibition zone became larger, indicating that the antimicrobial ability was enhanced. The crosslinked Schiff base enhanced the interaction between the cell wall peptidoglycan and the outer membrane lipoprotein, while the hydrophobic side formed by the coupling of the amine group with the aromatic aldehyde group of CIN reduced the adhesion of microbial cells on the surface (17). Moreover, different microbial cell structures between Gram-positive and Gram-negative bacteria led to the better antimicrobial performance on the *S. aureus*. The outer hydrophilic membrane of Gram-negative bacteria consists of lipopolysaccharide molecules that effectively block the hydrophobic compounds (60). CIN killed microorganisms by directly attacking the phospholipid membrane or by binding to enzymes on the cell wall resulting in increased permeability and exudation of the cytoplasm (61). Hammer et al. (62) demonstrated that CIN reduced cell membrane polarity and increased its permeability depending on the concentration and time. Shen et al. (63) pointed out that CIN had more than one antimicrobial mechanism and some compounds infiltrated into the bacteria before the intracellular solution changed. Chen et al. (64) confirmed the different effects of different concentrations of CIN on cells, where low concentration affected cell division, medium concentration affected cell energy supply and biofilm synthesis, and high concentration destroyed normal cell metabolism.

**Table 2:** The inhibition zones of grasshopper protein (GP)-based composite films against *Staphylococcus aureus* and *Escherichia coli*.

**Samples**	***S. aureus*** **inhibition zone (mm)**	***E. coli*** **inhibition zone (mm)**
CIN0	–	–
CIN10	–	–
CIN20	23.24 ± 0.56	20.75 ± 0.39
CIN30	39.21 ± 0.72	29.87 ± 3.27

## Conclusion

The mechanical properties, water resistance, and barrier properties of the composite film were improved obviously after mixing SPI. The tensile test confirmed that the film had the best mechanical properties when the ratio of GP to SPI was 7:3. The mechanical properties and water resistance of the films were further improved with xylose added as a crosslinker to trigger the Maillard reaction. The addition of xylose above 10% produced the saturation effect that would not improve the performance of the film anymore. The film start to gain in antimicrobial activity against *E. coli* and *S. aureus* when the CIN amount reached 20% and the antimicrobial ability became stronger with the increase of the content. However, CIN as a crosslinker failed to achieve the double enhancement effect with xylose, which has a negative effect on the mechanical properties and thermal stability of the film. Overall, this novel insect protein-based composite film showed good potential to be utilized as an edible antimicrobial film for active packaging.

## Data Availability Statement

The original contributions presented in the study are included in the article/supplementary material, further inquiries can be directed to the corresponding authors.

## Author Contributions

ZZ: conceptualization, investigation, and writing. XZ: conceptualization, review, editing, and supervision. CF: resources, project administration, and funding acquisition. DW: data curation. All authors contributed to the article and approved the submitted version.

## Funding

The authors acknowledge the financial support provided by the National Natural Science Foundation of China (Grant No. 51802259), China Postdoctoral Science Foundation Funded Project (Grant No. 2019M663785), Xi'an Programs for Science and Technology Plan (Grant Nos. 2020KJRC0090 and 21XJZZ0045), the Promotion Program for Youth of Shaanxi University science and technology association (Grant No. 20190415), the Outstanding Chinese and Foreign Youth Exchange Program of China Association for Science and Technology (CAST) in 2019, the Opening Project of Shanxi Key Laboratory of Advanced Manufacturing Technology (Grant No. XJZZ202001), and the Scientific Research Project of Shaanxi Education Department (Grant No. 20JS108).

## Conflict of Interest

The authors declare that the research was conducted in the absence of any commercial or financial relationships that could be construed as a potential conflict of interest.

## Publisher's Note

All claims expressed in this article are solely those of the authors and do not necessarily represent those of their affiliated organizations, or those of the publisher, the editors and the reviewers. Any product that may be evaluated in this article, or claim that may be made by its manufacturer, is not guaranteed or endorsed by the publisher.
